# Women in the 2019 hepatitis C cascade of care: findings from the British Columbia Hepatitis Testers cohort study

**DOI:** 10.1186/s12905-021-01470-7

**Published:** 2021-09-13

**Authors:** Margo E. Pearce, Sofia R. Bartlett, Amanda Yu, Jess Lamb, Cheryl Reitz, Stanley Wong, Maria Alvarez, Mawuena Binka, Héctor Velásquez Garcia, Dahn Jeong, Emilia Clementi, Prince Adu, Hasina Samji, Jason Wong, Jane Buxton, Eric Yoshida, Chelsea Elwood, Laura Sauve, Neora Pick, Mel Krajden, Naveed Z. Janjua

**Affiliations:** 1 British Columbia Centre for Disease Control, Vancouver, BC Canada; 2 School of Population and Public Health, University of British Columbia (UBC), Vancouver, BC Canada; 3 Department of Pathology and Laboratory Medicine, UBC, Vancouver, BC Canada; 4 AIDS Network Kootenay Outreach and Support Society, Kimberly, BC Canada; 5 East Kootenay Network of People who Use Drugs, Kimberly, BC Canada; 6 British Columbia Hepatitis Network Society, Vancouver, BC Canada; 7 Division of Gastroenterology, Department of Medicine, UBC, Vancouver, BC Canada; 8 Vancouver General Hospital, Vancouver, BC Canada; 9 Department of Obstetrics and Gynecology, UBC, Vancouver, Canada; 10 BC Women’s Hospital Research Institute, Vancouver, BC Canada; 11 Division of Infectious Diseases, Department of Pediatrics, UBC, Vancouver, Canada; 12 BC Children’s Hospital Research Institute, Vancouver, BC Canada; 13 Division of Infectious Diseases, Department of Medicine, UBC, Vancouver, BC Canada

## Abstract

**Background:**

Women living with hepatitis C virus (HCV) are rarely addressed in research and may be overrepresented within key populations requiring additional support to access HCV care and treatment. We constructed the HCV care cascade among people diagnosed with HCV in British Columbia, Canada, as of 2019 to compare progress in care and treatment and to assess sex/gender gaps in HCV treatment access.

**Methods:**

The BC Hepatitis Testers Cohort includes 1.7 million people who tested for HCV, HIV, reported cases of hepatitis B, and active tuberculosis in BC from 2000 to 2019. Test results were linked to medical visits, hospitalizations, cancers, prescription drugs, and mortality data. Six HCV care cascade stages were identified: (1) antibody diagnosed; (2) RNA tested; (3) RNA positive; (4) genotyped; (5) initiated treatment; and (6) achieved sustained virologic response (SVR). HCV care cascade results were assessed for women, and an ‘inverse’ cascade was created to assess gaps, including not being RNA tested, genotyped, or treatment initiated, stratified by sex.

**Results:**

In 2019, 52,638 people with known sex were anti-HCV positive in BC; 37% (19,522) were women. Confirmatory RNA tests were received by 86% (16,797/19,522) of anti-HCV positive women and 83% (27,353/33,116) of men. Among people who had been genotyped, 68% (6756/10,008) of women and 67% (12,640/18,828) of men initiated treatment, with 94% (5023/5364) of women and 92% (9147/9897) of men achieving SVR. Among the 3252 women and 6188 men not yet treated, higher proportions of women compared to men were born after 1975 (30% vs. 21%), had a mental health diagnosis (42% vs. 34%) and had used injection drugs (50% vs. 45%). Among 1619 women and 2780 men who had used injection drugs and were not yet treated, higher proportions of women than men used stimulants (64% vs. 57%), and opiates (67% vs. 60%).

**Conclusions:**

Women and men appear to be equally engaged into the HCV care cascade; however, women with concurrent social and health conditions are being left behind. Treatment access may be improved with approaches that meet the needs of younger women, those with mental health diagnoses, and women who use drugs.

**Supplementary Information:**

The online version contains supplementary material available at 10.1186/s12905-021-01470-7.

## Introduction

The treatment experiences and needs of women living with hepatitis C virus (HCV) are frequently overlooked in research, yet there are relevant clinical differences between men and women related to HCV infection and disease progression. Female sex is a significant predictor for spontaneous clearance among people with acute HCV infection and a factor in liver disease progression among those living with chronic HCV [1]. Rates of liver fibrosis and cirrhosis progression appear to be slower in younger women (< 50 years) compared to men; however, this difference disappears in older women (> 50 years), possibly due to hormonal changes in menopause [1, 2]. For women who have reproductive potential, HCV in pregnancy is a concern. Though pregnancy does not exacerbate HCV disease progression, HCV infection can contribute to adverse perinatal outcomes [3–6]. In addition, vertical HCV transmission affects 4–7% of infants born to women living with chronic HCV and up to 11% of infants born to women with HIV-HCV co-infection [7]. There are also gendered differences that underscore intersectional barriers faced by some women living with HCV. A cohort study in Ontario, Canada, highlighted that compared to men, women’s immigration status and lower socioeconomic status were more likely to negatively affect HCV treatment uptake [2]. Other studies have reported barriers to women’s access to HCV treatment, including advanced age, rurality, injection drug use, and involvement in sex work [8–12].

The introduction of novel direct-acting antiviral (DAA) therapies for chronic HCV infection has dramatically changed the HCV treatment landscape. In contrast with the arduous and moderately effective interferon-based treatment, DAA therapies are a well-tolerated and highly effective cure, with > 95% of patients achieving sustained virological response (SVR) in just 8–12 weeks [13]. Reduced clinical barriers to HCV cure have inspired the possibility of eliminating HCV globally by 2030, which will require diagnosing > 80% of those living with HCV and treating 85% of those diagnosed with chronic infection [14]. To achieve this, new HCV infections and deaths related to HCV need to be addressed alongside DAA treatments through scaled-up harm reduction and linkage to liver care.

A powerful way to monitor progress toward HCV elimination goals is to evaluate the HCV care cascade at the population level by assessing progress through RNA testing, genotype testing, treatment initiation, and SVR stages. In British Columbia (BC), Canada, integrated population-level laboratory testing and health administration data has made this possible. A 2018 analysis demonstrated that women comprised 37% of the approximately 53,000 people living with HCV in BC and that similar proportions of men and women progressed through the stages of care [15]. However, little is understood regarding factors that influence women’s access to HCV care at the population level. These factors may compound existing barriers and create, or negatively contribute to, risk environments where women are “hardly reached” by health/social services and at greater risk for adverse health outcomes [16]. Monitoring of HCV diagnosis and care among women is thus critical both to achieve HCV elimination goals in BC and ensure that women receive timely and equitable access.

The objectives of this study were to: (a) construct the population-level HCV care cascade in BC stratified by sex from 2000 to 2019; (b) evaluate progress through the stages of the 2019 HCV care cascade for women and men living with HCV in BC; (c) characterize progress and highlight gaps in the HCV care cascade experienced by women living with HCV in BC.

## Methods

This study represents population data from BC, Canada, where all residents are registered for publicly funded health insurance via the Medical Services Plan (MSP). MSP is a single-payer system covering healthcare provided by fee-for-service practitioners including general practices, private laboratories, and other providers. Laboratory HCV testing for the entire province is centralized at the BC Centre for Disease Control Public Health Laboratory (BCCDC PHL) except for 5% of tests that are performed at regional labs, which send specimens that test positive to BCCDC PHL for confirmation. All prescriptions dispensed in BC are recorded within a central payer-agnostic system called PharmaNet.

HCV therapies are publicly funded in BC through the PharmaCare Limited Coverage Drug Program. Interferon-based combination therapies (Interferon/Ribavirin) for HCV treatment became available in 2000, and the more efficacious Pegylated interferon/Ribavirin therapy became available in May 2003 [17]. DAA treatments were available in BC in 2014 and became publicly funded in early 2015, though eligibility for public coverage was restricted to priority patients with fibrosis stage 2 (F2) or above (Metavir or equivalent) or extrahepatic manifestations. In March 2017, eligibility for public coverage expanded to people with comorbidities including HIV or hepatitis B (HBV) co-infection, diabetes, chronic kidney disease, co-existent liver disease, and women who were planning to become pregnant in the next 12 months [18]. Remaining restrictions for publicly funded DAA treatment were removed in BC in April 2018. HCV testing and treatment in BC is provided in various healthcare settings including primary, community, and specialized clinics. It is important to note that prior to January 2020, new HCV antibody positive tests required a follow-up EDTA blood sample for HCV RNA nucleic acid testing (NAT). As of January 2020, persons who are positive for anti-HCV antibodies will automatically be tested for HCV RNA by NAT if: (1) they are first-time antibody positive or (2) if they have not been tested by NAT before. HCV genotype testing is required to prescribe HCV treatment in BC.

This analysis uses data from the British Columbia Hepatitis Testers Cohort (BC-HTC) study. We have previously published on the BC-HTC construction and data linkage [19]. Briefly, BC-HTC includes all BC residents who ever tested for HCV or HIV, or were diagnosed with HBV, HCV, HIV, or active tuberculosis (TB) in BC between 1990 and 2015, linked with data on medical visits, hospitalizations, cancers, prescription drugs, and deaths. The laboratory, prescription, and mortality data were updated to 31 December 2019 to facilitate creation and assessment of the 2019 HCV care cascade (Additional file 1: Table S1). In this study, we refer to ‘women’ as people who were assigned female sex at birth. Although ‘woman’ also implies gender identity, this was not determinable in this study.

BC-HTC data are de-identified and analyzed anonymously; thus, informed consent was not required. Institutional ethics approval was provided by the University of British Columbia Research Ethics Board (H14-01649) and all research was carried out in accordance with relevant guidelines and regulations.

### Cascade of HCV care

Operational definitions for six stages of the HCV cascade of care are described in Additional file 1: Table S2. The stages were defined as: a) HCV diagnosed; (b) HCV RNA tested; (c) HCV RNA positive; (d) genotyped; (e) initiated antiviral treatment; and (f) sustained viral response (SVR). We applied these definitions to the data to estimate the number and proportion of women in each stage by the end of the year from 2000 to 2019. Focusing on the year 2019, we also applied these definitions to compare the number and proportion of both men and women at each stage. Next, we evaluated the 2019 cascade stages by demographic characteristics and comorbidity profiles of women who were diagnosed with HCV. Finally, to get a clearer understanding of gaps and leakage in the HCV care cascade among women compared to men, we report on the *inverse 2019 HCV care cascade*: the number and proportions of women and men who were diagnosed anti-HCV positive but did not advance to HCV RNA testing, genotype testing, or treatment initiation stages.

### Estimate of viraemia

The estimate of HCV RNA positive women in BC in 2019 was based on: (1) the number of untreated women whose last HCV RNA test on record is positive; (2) 75% [20] of those who were positive by antibody testing and had no HCV RNA or genotype testing done, as about 25% of antibody-positive people clear infection spontaneously; (3) 75% [21] of the untested and undiagnosed estimate; (4) those treated women determined not to have achieved SVR (the SVR rate calculated for treated women with available RNA test after treatment was used to estimate how many treated women with no available RNA test after treatment would fail to achieve SVR) [15].

### Demographic characteristics and comorbidity profiles

Demographic characteristics included birth cohort, ethnicity, social and material deprivation [22], and urbanicity. Ethnicity was derived using Onomap software, which identifies ethnicity using name network cultural/linguistic clustering techniques [23–25]. Onomap has been previously validated and used in demographic and health research [23, 24]. Onomap is prone to misclassifying people with anglicized names and those with mixed ethnicities [26]; however, our internal validation demonstrated that Onomap’s sensitivity and specificity relative to self-identified ethnicity was 93% and 98.6% for South Asian people, respectively, and 66.7% and 99.5% for East Asian people, respectively. Ethnic groups were therefore classified as South Asian, East Asian, and Other BC Residents. Comorbidity indicators were derived from MSP data containing physician fee-for-service billing and diagnostic codes, and hospitalization data for mental health diagnoses, problematic alcohol and drug use, cirrhosis, and decompensated cirrhosis (Additional file 1: Table S3).

Characteristics and comorbidities of people diagnosed HCV antibody-positive were stratified by sex as well as proportions of women and men at each stage of the HCV care cascade. Chi-squared tests were carried out to compare categorical variables between women and men. All analyses were conducted using SAS/STAT software version 9.4 and R version 3.4.3.

### Role of the funding source

The BC Centre for Disease Control supported construction of the BC-HTC to inform policy and program related to HCV in BC. The study’s funders had no role in study design, data analysis, data interpretation, or writing of the article.

### Patient and public involvement

In Spring 2020, study investigators engaged with a community-based HIV/HCV organization in BC and a group of women with lived experience (WWLE) of the HCV care cascade to prioritize lines of inquiry. Over the next eight weeks, through a collaborative, consensus-based process, we reviewed results with the group of WWLE to take into account their perspectives and feedback and ensure findings were interpreted in ways that were destigmatizing and relevant to communities. This work culminated in two open-access 90-minute webinars in Summer 2020 that focused on the WWLE’s reflections and policy recommendations in response to the study results [27].

## Results

### Women in the HCV care cascade 2000–2019

**Fig. 1:**
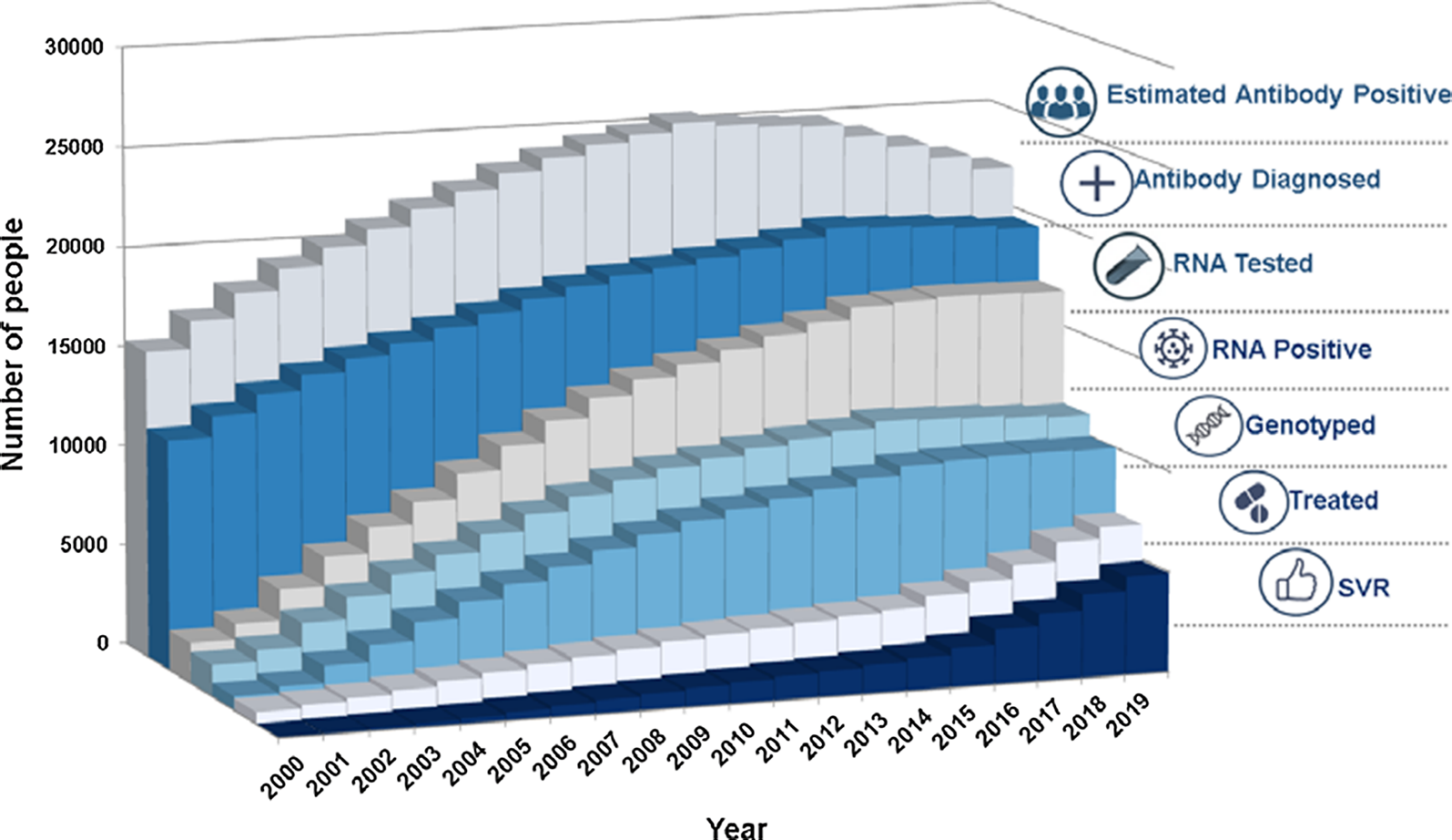
HCV care cascade from 2000–2019 for women diagnosed with HCV in British Columbia, Canada

Figure 1 displays women in the HCV cascade of care in BC from 2000 to 2019. The numbers of estimated and diagnosed anti-HCV positive women increased substantially from 2000 (15,305 and 11,479, respectively), reaching peaks in 2015 (24,710 and 20,062, respectively) followed by slight declines towards 2019 (22,056 and 19,522, respectively), which may be an artifact of the 2015 cohort data rather than fewer diagnoses. The number of antibody-positive women who underwent confirmatory HCV RNA testing also increased over time from 1987 (17.3%) in 2000 to 16,874 (84.9%) in 2017, followed by a slight decline to 16,797 (86%) in 2019. Coinciding with more women being HCV RNA tested, there was an increasing number of anti-HCV positive women with confirmed HCV RNA positive test results (i.e. chronic HCV infection) over the study period, from 1552 (78.1%) in 2000 to 11,075 (65.9%) in 2019. The number of women with chronic HCV infection who received a genotype test also increased substantially over the study period from 673 (43.4%) in 2000 to 10,007 (90.9%) in 2019. In 2000, just 572 of genotyped women initiated HCV treatment, and this number increased steadily over time, reaching 6755 (67.5%) in 2019. There was a corresponding increase in the number of women with known PCR information who achieved SVR after treatment, from 53% (279/531) in 2012 after Pegylated interferon/Ribavirin became available, to 79% (1,893/2,393) in 2015 when DAAs were offered to priority patients, to 94% (4,932/5,465) in 2019 when DAAs were available to all people in BC living with HCV.

### Estimated prevalence of anti-HCV positive women and viraemic proportion

The estimated prevalence of anti-HCV positive women in BC (including untested and undiagnosed women) declined from 25,251 (0.6%) in 2012 to 22,056 (0.4%) in 2019 (Additional file 1: Table S4). The estimated number of women who were viraemic also declined from 16,250 to 2012 (0.4%) to 8677 (0.2%) in 2019. The corresponding rate of viraemia declined from 64.4% of anti-HCV positive women in 2012 to 39.3% in 2019.

### The 2019 HCV care cascade for women and men in BC

**Fig. 2:**
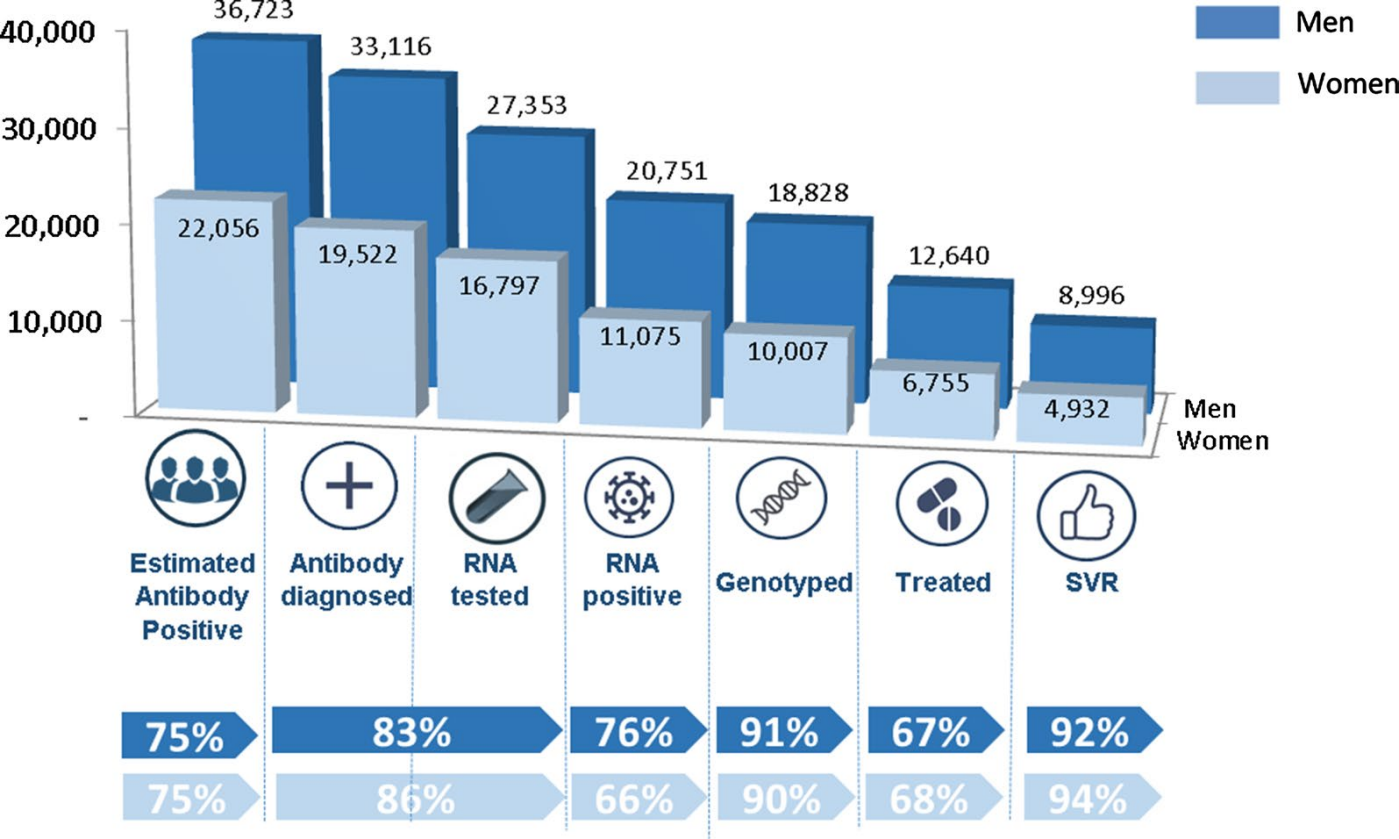
2019 HCV care cascade for women and men diagnosed with HCV in British Columbia, Canada

Figure 2 displays the 2019 HCV care cascade for women (light blue) and men (dark blue) in BC. In 2019, 52,638 people with known sex, including 19,522 women and 33,116 men were anti-HCV positive in 2019 (n = 3 people had unknown sex). The proportion of anti-HCV positive women receiving a confirmatory RNA test was 86% (16,797/19,522) compared to 82.6% (27,353/33,116) of men. 34% (5721/16,797) of women RNA tested had negative results (spontaneously cleared) compared to 24.1% (6602/27,353) of men. Among people who had the virus they acquired genotyped, 68% (6756/10,008) of women and 67% (12,640/18,828) of men initiated treatment, with 94% (5023/5364) of women and 92% (9147/9897) of men achieving SVR.

### Characteristics and comorbidity profiles of HCV antibody-positive women in 2019, stratified by cascade of care stages

Characteristics and comorbidity profiles of women at each stage of the 2019 HCV care cascade are displayed in Table 1. Women born within the 1945–1964 birth cohort represented the highest proportion of all anti-HCV positive women (51.5%) and in each stage of the care cascade thereafter, including 56.2% of those genotyped, 63.5% of those who initiated HCV treatment, and 66.5% of those who achieved SVR. Younger women born between 1965 and 1974 and ≥ 1975 were 21.3 and 22.2% of all anti-HCV positive women, respectively, yet smaller proportions of those women progressed through the care cascade, making up just 17.7 and 14% of women who initiated HCV treatment, and 16.5 and 12.4% of women who achieved SVR, respectively. Women with East Asian or South Asian ethnicity comprised 5.4 and 4.5% of anti-HCV positive women in BC, with proportions increasing slightly as the women progressed through the care cascade stages, reaching 5.6 and 5.9% of women who initiated HCV treatment and 6.1 and 5.3% of women who achieved SVR, respectively. Women within the most materially deprived quintile (Q5) made up a consistent proportion in each cascade stage, including 27.6% of those anti-HCV diagnosed, 27.8% of those RNA tested, 26.9% of those genotyped, 24.6% of those who initiated treatment, and 23.7% of those who achieved SVR. The proportions of women in the cascade stages who were in the most socially deprived quintile (Q5) increased from 22% of those anti-HCV diagnosed and RNA tested to 35.9% of those genotyped, 32.9% of those who initiated treatment, and 32.3% of those who achieved SVR.

**Table 1:** Demographic characteristics and comorbidity profile for women diagnosed with HCV in BC in 2019, stratified by care cascade stages

Variable	Antibody Diagnosed		HCV RNA Tested		HCV RNA Positive		HCV Genotyped		HCV Treatment Initiated		SVR Achieved/Unknown		SVR Achieved	
	n	%	n	%	n	%	n	%	n	%	n	%	n	%
Total	19,522	100	16,797	100	11,075	100	10,007	100	6755	100	5683	100	4932	100
*Birth Cohort*														
< 1945	974	4.99	786	4.68	570	5.15	505	5.05	322	4.77	270	4.75	228	4.62
1945–1964	10,046	51.46	8701	51.8	6028	54.43	5620	56.16	4288	63.48	3680	64.75	3279	66.48
1965–1974	4162	21.32	3492	20.79	2210	19.95	1975	19.74	1197	17.72	986	17.35	814	16.5
> 1974	4340	22.23	3818	22.73	2267	20.47	1907	19.06	948	14.03	747	13.14	611	12.39
*Ethnicity*														
East Asian	1045	5.35	864	5.14	542	4.89	496	4.96	379	5.61	331	5.82	302	6.12
South Asian	872	4.47	803	4.78	549	4.96	521	5.21	395	5.85	338	5.95	263	5.33
Other BC Residents	17,605	90.2	15,130	90.1	9984	90.15	8990	89.84	5981	88.54	5014	88.22	4367	88.55
*Material Deprivation Quintile*														
Q1 (most privileged)	2622	13.43	2294	13.66	1514	13.67	1390	13.89	953	14.11	797	14.02	698	14.15
Q2	2985	15.29	2602	15.49	1731	15.63	1597	15.96	1140	16.88	958	16.86	844	17.11
Q3	3557	18.22	3085	18.37	2058	18.58	1859	18.58	1337	19.79	1135	19.97	995	20.17
Q4	4407	22.57	3778	22.49	2511	22.67	2289	22.87	1569	23.23	1351	23.77	1155	23.42
Q5 (most deprived)	5391	27.61	4669	27.8	3033	27.39	2689	26.87	1662	24.6	1361	23.95	1168	23.68
Unknown	560	2.87	369	2.2	228	2.06	183	1.83	94	1.39	81	1.43	72	1.46
*Social Deprivation Quintile*														
Q1 (most privileged)	2351	12.04	369	2.2	1369	12.36	1258	12.57	923	13.66	766	13.48	642	13.02
Q2	2337	11.97	2053	12.22	1320	11.92	1209	12.08	878	13	768	13.51	648	13.14
Q3	2992	15.33	2018	12.01	1749	15.79	1591	15.9	1113	16.48	943	16.59	838	16.99
Q4	4332	22.19	2599	15.47	2392	21.6	2175	21.73	1522	22.53	1269	22.33	1137	23.05
Q5 (most deprived)	6950	35.60	3717	22.13	4017	36.27	3591	35.88	2225	32.94	1856	32.66	1595	32.34
Unknown	560	2.87	6041	35.96	228	2.06	183	1.83	94	1.39	81	1.43	72	1.46
*Urbanicity*														
Rural	2525	12.93	2222	13.23	1484	13.4	47	0.47	983	14.55	845	14.87	747	15.15
Urban	16683	85.46	14436	85.94	9511	85.88	1379	13.78	5766	85.36	4835	85.08	4182	84.79
Unknown	314	1.61	139	0.83	80	0.72	8581	85.75	6	0.09	3	0.05	3	0.06
*History of Injection Drug Use*														
Yes	7265	37.21	6497	38.68	4210	38.01	3741	37.38	2122	31.41	1698	29.88	1412	28.63
No	12257	62.79	10300	61.32	6865	61.99	6266	62.62	4633	68.59	3985	70.12	3520	71.37
History of Opioid use*														
Yes	4556	62.71	4154	63.94	2654	63.04	2375	63.49	1294	60.98	1021	60.13	843	59.7
No	2709	37.29	2343	3606	1556	36.96	1366	36.51	828	39.02	677	39.87	569	40.3
History of stimulant use*														
Yes	4327	59.56	3938	60.61	2513	59.69	2238	59.82	1202	56.64	945	55.65	768	54.39
No	2938	40.44	2559	39.39	1697	40.31	1503	40.18	920	43.36	753	44.35	644	45.61
*Problematic Alcohol Use*														
Yes	4952	25.37	4442	26.45	2797	25.26	2534	25.32	1549	22.93	1284	22.59	1084	21.98
No/Unknown	14570	74.63	12355	73.55	8278	74.74	7473	74.68	5206	77.07	4399	77.41	3848	78.02
*Major Mental Health Diagnosis*														
Yes	7,379	37.8	6646	39.57	4331	39.11	3945	39.42	2572	38.08	2115	37.22	1804	36.58
No/Unknown	12,143	62.2	10,151	60.43	6744	60.89	6062	60.58	4183	61.92	3568	62.78	3128	63.42
*HBV Coinfection*														
Yes	905	4.64	904	5.38	579	5.23	538	5.38	366	5.42	575	5.42	487	9.87
No/Unknown	18,617	95.36	15,893	94.62	10,496	94.77	9469	94.62	6389	94.58	5108	94.58	4445	90.13
*HIV/AIDS Coinfection*														
Yes	565	2.89	904	5.38	475	4.29	448	4.48	281	4.16	233	4.1	204	4.14
No/Unknown	18,957	97.11	16,096	95.82	10,600	95.72	9559	95.53	6474	95.84	5450	95.9	4728	95.86
*Cirrhosis*														
Yes	807	4.13	752	4.48	608	5.49	581	5.81	493	7.3	418	7.36	379	7.68
No/Unknown	18,715	95.87	16,045	95.52	10,467	94.51	9426	94.19	6262	92.7	5265	92.64	4553	92.32
*Decompensated Cirrhosis*														
Yes	468	2.4	423	2.52	331	2.99	308	3.08	245	3.63	205	3.61	187	3.79
No/Unknown	19,054	97.6	16,374	97.48	10,744	97.01	9699	96.92	6510	96.37	5478	96.39	4745	96.21

*Includes only women with a history of injection drug use

Women with a history of injection drug use made up 37.2% of women who were anti-HCV positive, 38.7% of women who were RNA tested, and 37.4% of women who were genotype tested, respectively; yet, they made up just 31.4% of women who initiated treatment and 28.6% of women who achieved SVR. Women with a history of injection drug use and who had used opioids made up about two-thirds of women in each stage of the HCV care cascade. Women with a history of injection drug use and who had used stimulants made up 59.5% of anti-HCV positive women, 60.6% of those RNA tested, 59.8% of those genotyped, and 56.6% of those initiated on treatment. The proportions of women in each stage of the cascade who had been diagnosed with a major mental health disorder were fairly consistent, including 37.8% of those anti-HCV positive, 39.6% of those RNA tested, 39.4% of those genotyped, 38.1% of those who started treatment, and 36.6% of those who achieved SVR. Women with HCV/HBV co-infection made up 4.6% of women who were anti-HCV positive, 4.3% of those RNA tested and genotyped, 4.4% of those treatment initiated, and 4.6% of those who achieved SVR. Women with HCV/HIV co-infection made up 2.9% of anti-HCV positive women, were 3.1% of those who were RNA tested, and 3.3% of those genotyped, 3% of those treatment initiated, and 3% of those who achieved SVR. Women with liver cirrhosis comprised 4.1% of anti-HCV positive females, and made up somewhat increasing proportions of those who were RNA tested (5.5%), genotyped (5.8%), initiated treatment 7.3%, and achieved SVR (7.7%).

### Characteristics and comorbidity profiles of antibody diagnosed men and women in 2019 in BC, stratified by inverse HCV care cascade stages (not RNA tested, not genotyped, not treated)

Characteristics and comorbidity profiles of men and women within the inverse 2019 HCV care cascade are presented in Table 2. Women born between 1945 and 1974 made up the majority of women who were not RNA tested (49.4%), genotyped (38.2%), or initiated on treatment (41%); yet, men within the 1945–1974 birth cohort made up relatively greater proportions of men who were not RNA tested (58.7%), genotyped (49.6%), or initiated on treatment (49.1%). Higher proportions of younger anti-HCV positive women born ≥ 1975 than men were not RNA tested (19.2% vs. 11.9%, respectively), genotyped (33.7% vs. 20.2%, respectively), or initiated on treatment (29.5% vs. 20.8%, respectively). A higher proportion of anti-HCV positive East Asian women compared to East Asian men had not been RNA tested (6.6% vs. 3.3%, respectively) (*p* < 0.001), though similar proportions had not been genotyped (4.3% of women vs. 3.4% of men) or initiated on treatment (3.6% of women vs. 2% of men). Higher proportions of anti-HCV positive women than men who had not been RNA tested were within the most deprived material quintiles (*p* = 0.034) whereas lower proportions of women than men who had not been RNA tested were within the most deprived social quintiles (*p* = 0.013).

**Table 2:** Demographic characteristics and comorbidity profile for women diagnosed with HCV in BC in 2019, who did not progress to the next stage of HCV care

	Anti-HCV positive					Not HCV RNA tested					Not genotyped	
	Women		Men			Women		Men			Women	
	n	%	n	%	*p-value*	n	%	n	%	*p-value*	n	%
Total	19,522	100	33,116	100		2725	100	5763	100		1068	100
*Birth Cohort*												
<1945	974	5	1151	3.48	<0.001	188	6.9	256	4.44	<0.001	65	6.09
1945-1964	10,046	51.46	19,797	59.78		1345	49.36	3382	58.68		408	38.2
1965-1974	4162	21.32	7374	22.27		670	24.59	1440	24.99		235	22
≥1975	4340	22.23	4794	14.48		522	19.16	685	11.89		360	33.71
*Ethnicity*												
East Asian	1045	5.35	1128	3.41	<0.001	181	6.64	190	3.3	<0.001	46	4.31
South Asian	872	4.47	1283	3.87		69	2.53	128	2.22		28	2.62
Other BC Residents	17,605	90.2	30,705	92.72		2475	90.82	5445	94.48		994	93.07
*Material deprivation quintile*												
Q1 (most privileged)	2622	13.43	4912	14.83	<0.001	328	12.04	777	13.48	0.034	124	11.61
Q2	2985	15.29	5018	15.15		383	14.06	812	14.09		134	12.55
Q3	3557	18.22	5851	17.67		472	17.32	905	15.7		199	18.63
Q4	4407	22.57	7025	21.21		629	23.08	1165	20.22		222	20.79
Q5 (most deprived)	5391	27.61	8972	27.09		722	26.5	1474	25.58		344	32.21
Unknown	560	2.87	1338	4.04		191	7.01	630	10.93		45	4.21
*Social deprivation quintile*												
Q1 (most privileged)	2351	12.04	3643	11	<0.001	298	10.94	537	9.32	0.013	111	10.39
Q2	2337	11.97	3955	11.94		319	11.71	612	10.62		111	10.39
Q3	2992	15.33	4791	14.47		393	14.42	751	13.03		158	14.79
Q4	4332	22.19	6877	20.77		615	22.57	1181	20.49		217	20.32
Q5 (most deprived)	6950	35.6	12,512	37.78		909	33.36	2052	35.61		426	39.89
Unknown	560	2.87	1338	4.04		191	7.01	630	10.93		45	4.21
*Urbanicity*												
Rural	2525	12.93	4396	13.27	0.077	303	11.12	646	11.21	0.453	105	9.83
Urban	16,683	85.46	27,699	83.64		2247	82.46	4531	78.62		930	87.08
Unknown	314	1.61	1021	3.08		175	6.42	586	10.17		33	3.09
*History of Injection Drug Use*												
Yes	7265	37.21	11,005	33.23	<0.001	768	28.18	1391	24.14	<0.001	469	43.91
No	12,257	62.79	22,111	66.77		1957	71.82	4372	75.86		599	56.09
History of Opioid use*												
Yes	4556	62.71	6370	57.88	<0.001	402	47.66	688	49.46	0.200	279	59.49
No	2709	37.29	4635	42.12		366	52.34	703	50.54		190	40.51
History of stimulant use*												
Yes	4327	59.56	6023	54.73	<0.001	389	50.65	706	50.75	0.963	275	58.64
No	2938	40.44	4982	45.27		379	49.35	685	49.25		194	41.36
*Problematic Alcohol Use*												
Yes	4952	25.37	8809	26.6	0.002	510	18.72	1172	20.34	0.080	263	24.63
No/Unknown	14,570	74.63	24,307	73.4		2215	81.28	4591	79.66		805	75.37
*Major Mental Health Diagnosis*												
Yes	7379	37.8	9527	28.77	<0.001	733	26.9	1101	19.1	<0.001	386	36.14
No/Unknown	12,143	62.2	23,589	71.23		1992	73.1	4662	80.9		682	63.86
*HBV Coinfection*												
Yes	905	4.64	1968	5.94	0.288	183	6.72	395	6.85	0.933	41	3.84
No/Unknown	18,617	95.36	31,148	94.05		2542	93.29	5368	93.14		1027	96.16
*HIV/AIDS Coinfection*												
Yes	565	2.89	1826	5.51	<0.001	61	2.24	200	3.47	0.014	27	2.53
No/Unknown	18,957	97.11	31,290	94.48		2664	97.76	5563	96.53		1041	97.47
*Cirrhosis*												
Yes	807	4.13	1474	4.45	0.084	55	2.02	115	2	0.944	27	2.53
No/Unknown	18,715	95.87	31,642	95.55		2670	97.98	5648	98		1041	97.47
*Decompensated Cirrhosis*												
Yes	468	2.4	776	2.34	0.694	45	1.65	78	1.35	0.284	23	2.15
No/Unknown	19,054	97.6	32,340	97.66		2680	98.35	5685	98.65		1045	97.85

	Not Genotyped			Not treatment initiated				
	Men			Women		Men		
	n	%	*p-value*	n	%	n	%	*p-value*
Total	1923	100		3252	100	6188	100	
*Birth Cohort*								
<1945	75	3.9	<0.001	183	5.63	195	3.15	<0.001
1945-1964	953	49.56		1332	40.96	3038	49.1	
1965-1974	507	26.37		778	23.92	1671	27	
≥1975	388	20.18		959	29.49	1284	20.75	
*Ethnicity*								
East Asian	65	3.38	0.621	117	3.6	124	2	<0.001
South Asian	55	2.86		126	3.87	207	3.35	
Other BC Residents	1803	93.76		3009	92.53	5857	94.65	
*Material Deprivation Quintile*								
Q1 (most privileged)	244	12.69	0.825	437	13.44	891	14.4	0.417
Q2	236	12.27		457	14.05	795	12.85	
Q3	329	17.11		522	16.05	1009	16.31	
Q4	395	20.54		720	22.14	1348	21.78	
Q5 (most deprived)	613	31.88		1027	31.58	1957	31.63	
Unknown	106	5.51		89	2.74	188	3.04	
*Social Deprivation Quintile*								
Q1 (most privileged)	189	9.83	0.946	335	10.3	587	9.49	0.064
Q2	203	10.56		331	10.18	648	10.47	
Q3	267	13.88		478	14.7	809	13.07	
Q4	377	19.6		653	20.08	1208	19.52	
Q5 (most deprived)	781	40.61		1366	42	2748	44.41	
Unknown	106	5.51		89	2.74	188	3.04	
*Urbanicity*								
Rural	209	10.87	0.305	396	12.18	702	11.34	0.285
Urban	1626	84.56		2815	86.56	5361	86.64	
Unknown	88	4.58		41	1.26	125	2.02	
*History of Injection Drug Use*								
Yes	714	37.13	0.003	1619	49.78	2780	44.93	<0.001
No	1209	62.87		1633	50.22	3408	55.07	
History of Opioid use*								
Yes	380	53.22	0.034	1081	66.77	1656	59.57	<0.001
No	334	46.78		538	33.23	1124	40.43	
History of stimulant use*								
Yes	399	54.34	0.146	1036	63.99	1580	56.83	<0.001
No	326	45.66		583	36.01	1200	43.17	
*Problematic Alcohol Use*								
Yes	540	28.08	0.041	985	30.29	2051	33.14	0.005
No/Unknown	1383	71.92		2267	69.71	4137	66.86	
*Major Mental Health Diagnosis*								
Yes	567	29.49	0.002	1373	42.22	2105	34.02	<0.001
No/Unknown	1356	70.51		1879	57.78	4083	65.98	
*HBV Coinfection*								
Yes	82	4.26	0.729	172	5.29	266	4.3	0.084
No/Unknown	1841	95.74		3080	94.71	5922	95.7	
*HIV/AIDS Coinfection*								
Yes	43	2.24	0.793	167	5.14	313	5.06	0.971
No/Unknown	1880	97.77		3085	94.87	5875	94.94	
*Cirrhosis*								
Yes	46	2.39	0.817	88	2.71	185	2.99	0.435
No/Unknown	1877	97.61		3164	97.29	6003	97.01	
*Decompensated Cirrhosis*								
Yes	30	1.56	0.239	63	1.94	112	1.81	0.663
No/Unknown	1893	98.44		3189	98.06	6076	98.19	

*CI* confidence interval, *HR* Hazard ratio. Multivariable analysis included adjustment for sex, Dukes class for test series and TNM4-stage for validation series, differentiation grade (G1–2 vs. G3–4), and age (as continuous). Age and TNM-stage/Dukes-classification also remained as independent predictors of prognosis in the multivariable mode

Increasing proportions of women and men who used injection drugs were left behind in the HCV cascade of care stages. Higher proportions of women than men who had injected drugs had not been RNA tested (28.2% vs. 24.1%, respectively) (*p* < 0.001), genotyped (43.9% vs. 37.1%, respectively) (*p* < 0.001), or treated (49.8% vs. 44.9%, respectively). (*p* < 0.001) Corresponding disparities were observed among people with a history of injection drug use who had used opioids, with 59.5% of women compared to 53.2% of men not being genotyped (*p* < 0.034), and 66.8% of women compared to 59.6% of men not initiating treatment (*p* < 0.001). Among people with a history of injection drug use who had used stimulants, 58.6% of women compared to 54.3% of men had not been genotyped and 64% of women compared to 56.8% of men had not initiated treatment (*p* < 0.001). Higher proportions of anti-HCV diagnosed women with a mental health diagnosis compared to men with a mental health diagnosis had not been RNA tested (27% vs. 19.1%, respectively) (*p* < 0.001), genotyped (36.1% vs. 29.5%, respectively) (*p* < 0.001), or initiated on treatment (42.2% vs. 34%, respectively) (*p* < 0.001).

## Discussion

Using population-based HCV care cascade monitoring data, this study has described women within and outside the HCV care cascade in BC, Canada. We observed steady progress across the care cascade among women living with HCV, with a substantial increase in treatment uptake after the introduction of DAAs in 2015 and expanded coverage starting in 2017. This increase also led to a reduction in the estimated prevalence of viraemic women in the province, from 0.4% to 2000 to 0.2% in 2019 [15]. In 2019, nearly equal proportions of women and men progressed through the HCV care cascade. These results should encourage public health programming and treatment providers that significant progress is being made to eliminate HCV infection in BC. We also identified key groups of women being left behind in the care cascade: specifically, younger women were less likely to progress across the cascade stages compared to men of the same age, which may impact reproductive outcomes. Similarly, women with problematic substance use were less likely to receive treatment. These findings highlighted opportunities to adapt programming and clinical care plans to accommodate women’s needs, as HCV risk environments and barriers to treatment frequently intersect with sex and gender-based realities.

This study demonstrated that in 2019, just over half of women diagnosed anti-HCV positive in BC were born between 1945 and 1964 (baby boomers) and that this birth cohort represented an increasing proportion of women in subsequent HCV care cascade stages. Women in the 1965–1974 birth cohort comprised a significant proportion of women who were RNA positive and of those treated for HCV. These findings support previous research demonstrating that though the overall rate of HCV infection in the 1945–1974 birth cohort is declining, this population still makes up the majority of prevalent HCV infections in BC and Canada and those in need of HCV treatment [14, 28, 29]. Baby boomers have thus been identified as a priority population in Canada’s HCV elimination targets, and national testing guidelines are for one-time HCV screening of all Canadians born between 1945–1974 [14, 30]. Most HCV infections among baby boomers result from past exposure in medical settings or past injection drug use; however, they may be less likely to seek out testing and treatment due to a lack of HCV awareness, difficulty recalling past exposures, or stigma related to substance use [31]. In the inverse HCV care cascade, women and men born between 1945 and 1964 made up similarly higher proportions of those not RNA tested, genotyped, or treatment initiated. Women born between 1965 and 1974 made up about one quarter of women not RNA tested, genotyped, or treatment initiated. As previously discussed, for older women living with HCV, the risk for accelerated liver fibrosis progression becomes a concern. Most younger women living with chronic HCV experience slower liver disease progression, including cirrhosis and hepatocellular carcinoma[32], but some biological studies suggest that post-menopausal women may lose the putative protective effect of estrogen on the liver due to a decline of estrogen levels in the post-menopausal period [1, 33]. Older women who have unknowingly been living with HCV for decades and those who are aware of their HCV diagnosis but have not yet engaged in the HCV care cascade may be at risk for advanced liver disease. Promising interventions aimed at increasing HCV screening and linkage to HCV care among baby boomers have leveraged the utility of electronic health records by adding HCV status to routine patient maintenance reminders for healthcare providers, followed by coordinated linkage to HCV treatment [31]. Similar approaches that also work to reduce the stigma associated with HCV infection may serve to identify older women in BC who are unaware of their HCV status and encourage engagement in the care cascade [11].

Younger women born after 1975 comprised 22.2% of anti-HCV positive women, yet made up successively lower proportions of women in each HCV care cascade stage in 2019. Conversely, in the inverse HCV care cascade, these women comprised higher proportions than men among those not RNA tested, genotyped, or initiated on treatment. This finding parallels a previous study using population laboratory surveillance data in BC that demonstrated a significant increase over time in the proportion of newly diagnosed HCV positive women within an age range of reproductive potential who were lost to follow-up for RNA and/or genotype testing – from 10.2% to 2008 to 24.3% in 2019 [34]. Similarly, a large cohort study involving Veterans Administration data in the United States that found younger women had significantly lower odds of receiving DAA treatment than younger men [35]. As mentioned above, there is risk for vertical transmission among younger women living with HCV who become pregnant. A number of population-based studies in the US have indicated that rising maternal and pediatric HCV prevalence is likely related to concomitant increasing opioid use among women of reproductive potential [3, 36–38]. In the BC Hepatitis Testers Cohort, 61% of women with chronic HCV infection who were born after 1975 had histories of injection drug use and opiate use, among whom 50% had not been treated for HCV as of 2019. Treating women before or between pregnancies is therefore essential, yet, considering gendered realities faced by women living with HCV, they must be assured that they will receive individual and family support as part of their HCV care plan. Younger women with past or current substance use may avoid or delay both prenatal and HCV care because of potential stigma within healthcare towards people who use substances or the possibility of their children being apprehended due to child welfare concerns [39]. Younger women may also be managing competing health, social, and economic priorities and feel they must delay treatment [40]. Outside of pregnancy, HCV infection is a concern for women’s health [41]. Because of younger women’s typical slower progression of liver disease, healthcare providers may mistakenly not prioritize HCV treatment for younger women with chronic HCV infection. Awareness of the potential long and short-term extrahepatic manifestations of HCV infection and potential improvements in quality of life should be emphasized to both care providers and younger women living with HCV.

The majority of anti-HCV positive women in each stage of the HCV care cascade were within the most severe quintiles for material and social deprivation. In the inverse HCV care cascade, similarly high proportions of anti-HCV positive women and men who were not RNA tested, genotyped, or initiated on treatment had severe material deprivation. Likewise, high and similar proportions of anti-HCV positive women and men who had not been RNA tested, genotyped, or initiated on treatment had severe social deprivation. Though HCV care and treatment in BC is publically available to all living with HCV through universal healthcare, poverty and social isolation intersect with multifaceted issues faced by women with HCV. Women living with HCV have reported navigating gender-based violence, racism in the healthcare system, and immigration processes while juggling work, childcare, and other competing priorities [10, 42, 43]. These situations can create complex obstacles to women’s HCV care, wherein some groups of women become among those who are “hardly reached” by treatment providers [15, 16]. It is important to note that although HCV positive women who have recently immigrated, who are Indigenous or Black, are involved in sex work, or unstably housed were not identifiable in our study, the experiences and healthcare needs of these key groups of women have been previously highlighted in research and must not be overlooked moving forward [10, 43, 44]. Awareness of barriers and expansion of specialized, women-centered approaches, such as culturally-safe HCV outreach and peer-support programming, are therefore essential [45].

Women in BC with a history of injection drug use made up 37.2% of women who were HCV antibody diagnosed, 38.7% who were RNA tested, and 37.4% who were genotype tested; yet, they made up just 31.4% of women who initiated treatment and 28.6% of women who achieved SVR. In the inverse HCV care cascade, somewhat higher proportions of women compared to men who not been RNA tested, genotyped, or initiated on treatment had injected drugs. The proportions of both women and men living with HCV and a history of injection drug use and opioid or stimulant use steadily increased across the inverse cascade stages. Nevertheless, disproportionately higher numbers of anti-HCV positive women who had used opioids or stimulants were left behind in the care cascade. These findings correspond to studies based in the United States that have reported a high frequency of opiate and stimulant use among women at risk of or living with HCV [46]. In other BC population-based analyses, uninterrupted opioid agonist therapy (OAT) was associated with higher likelihood HCV treatment uptake among people who inject drugs after adjusting for sex, yet stimulant use disorder was negatively associated with treatment uptake [47, 48]. Research has also demonstrated that gendered power dynamics contribute higher HCV exposure risk for women, such as being second on the needle, requiring help to inject, and needing to negotiate harm reduction with risk for violence [49–51]. Women with lived experience of HCV have highlighted that intersecting experiences of sexism, racism, and discrimination toward women who use injection drugs create significant barriers to accessing healthcare, including addiction treatment [27, 43]. Involving HCV-affected women who use drugs in the design and delivery of HCV screening, treatment, and harm reduction programming will result in innovative solutions that address these barriers and lead to more women engaging in the HCV care cascade and experiencing improved wellbeing beyond achieving SVR.

Overall, this study demonstrated that in 2019, 37.8% of women and 28.8% of men who were diagnosed anti-HCV positive in BC had had a mental health diagnosis. Anti-HCV positive women with a mental health disorder made up about 40% of women within each stage of the HCV care cascade and increasing proportions of women in each inverse HCV care cascade stage. Higher proportions of women compared to men who had not been RNA tested, genotyped, or initiated on treatment had received a mental health diagnosis. National self-reported data suggests that women in Canada are more likely than men to have had past and recent major depression and generalized anxiety [52] and more likely to perceive that their mental health care needs are not met [53]. Intervention research based in the United States and Australia has reported that patients with severe mental health diagnoses who received HCV care integrated with mental health care had a higher likelihood of achieving SVR [54]. Few of the study participants were women, however, and therefore the relevance and effectiveness of such interventions for women who have mental illness and are living with HCV is unclear. In addition, mental health disorders are frequently concurrent with problematic substance use, requiring specialized care and harm reduction. Women-centred HCV interventions that are trauma-informed, culturally safe, and work within peer-support frameworks may better meet the needs of women diagnosed with mental health disorders [55].

We found that the proportion of women in each stage of the 2019 HCV care cascade living with HCV-HBV co-infection was relatively constant at about 4.5%. In the inverse cascade, proportions of women and men with HCV-HBV co-infection who were not RNA tested were somewhat higher than the proportions who were not genotyped or initiated on treatment, highlighting that those who received RNA testing are likely to progress through subsequent HCV care cascade stages. Somewhat higher proportions of men compared to women in the inverse cascade stages were living with HCV-HIV co-infection, likely reflecting the higher burden of HIV infection among men in BC. Proportions of women and men living with HCV who had cirrhosis and decompensated cirrhosis were similar. It is important to note that although more prevalent among men living with HCV, over 25% of women not HCV RNA tested, genotyped, or treated had problematic alcohol use. Accelerated liver disease progression among these women is of grave concern, especially among those unaware of their HCV diagnosis or treatment options. Continued focus on providing HCV treatment to women living with significant comorbidities is needed, specifically with enhanced models that address relational and contextual barriers to engaging in healthcare among women with HCV and HBV or HIV co-infection.

## Limitations

Although this study is based on comprehensive data to characterize the HCV cascade of care in BC, there are limitations that impact the measurement of each stage. The model to estimate the number of people who were undiagnosed HCV antibody-positive was based on 2012 BC and Canadian data [21, 56]. BC residents have historically tested for HCV more than other provinces, with testing volumes increasing in recent years, especially after the STOP-HIV initiative began in BC, suggesting that our estimate of the proportion who are undiagnosed may be lower than the national average. Further, the national mandate to test all baby boomers for HCV has increased the number of people born between 1945 and 1965 living with HCV infection who have been diagnosed; subsequently, HCV positivity is declining in this age group. Simultaneously, the number of new/incident cases of HCV have fallen in BC over the past decade, mortality among people with chronic HCV is higher compared to people without HCV, and uptake of curative DAA treatments is increasing [57]. This study may therefore overestimate the number of undiagnosed and prevalent cases of HCV in BC; however, the estimated fraction of undiagnosed people in our cohort was similar to what Hamadeh et al. (2020) reported in population model estimates of chronic HCV infection in the province (33.3%) [58]. In addition, BC-HTC data does not contain information about gender identity, and therefore we cannot comment on the HCV care cascade experienced by people classified as female sex assigned at birth but who do not identify as women. We recognize that transgender men and other gender-diverse people may experience unique barriers to HCV screening and linkage to HCV care. Future work should focus on the specific HCV care needs of this key population. Though we validated Onomap for use in the BC population, it is not able to identify all people, in particular: those who would describe themselves as having a mixed ethnicity; people whose surnames are not specific to ethnic groups, and; people who adopt surnames of another ethnic group. Onomap does not identify people with Indigenous ethnicity. Due to legislated forced assimilation in Canada, many Indigenous peoples’ names were changed to biblical or other European names [59]. Thus, there is a misclassification of various ethnic groups through this methodology. We used diagnostic codes in administrative datasets to assess history of mental illness and substance use. This raises several issues: bias towards underestimating prevalence in those less engaged in healthcare, and potential misclassification related to sensitivity and specificity of these measures. Potentially lower linkage rates in some key groups would result in less representation, especially people who are homeless, street-involved, and incarcerated [19].

## Conclusions

This study has shown that women are progressing similarly to men across the HCV care cascade stages. However, gaps remain for some groups of women, particularly baby boomers and younger women, women experiencing poverty and social isolation, women with problematic substance use, and women with mental health disorders. Though access to HCV testing and treatment has expanded dramatically with DAAs, systemic barriers to testing and treatment in BC, especially within primary care and community-based health and social services [60], disproportionately impact marginalized populations. Programming that is peer-based and specifically reaches out to support women to engage or re-engage with the HCV care cascade could help BC reach HCV elimination targets, as well as achieve equity of health care access and outcomes. Such programming must understand and address the overlapping challenges faced by women living with HCV, as they are frequently gendered and exacerbate barriers to engaging in any form of healthcare.

## Supplementary Information

**Additional file 1**. Supplementary Materials.

## Data Availability

All data generated or analysed during this study are included in this published article [and its supplementary information files].
