# Supplementary material for: Women in the 2019 hepatitis C cascade of care: findings from the British Columbia Hepatitis Testers cohort study
Source: BMC Womens Health. 2021 Sep 13;21:330. doi: 10.1186/s12905-021-01470-7 (PMC8436483; doi:10.1186/s12905-021-01470-7)
Supplement: Supplementary file 1 — Additional file 1. Supplementary Materials. [file 12905_2021_1470_MOESM1_ESM.docx]

**Additional file 1: Table S1. Criteria and Data Sources for the BC Hepatitis Testers Cohort (BC-HTC)
Web:** [**https://bchtc.med.ubc.ca/**](https://bchtc.med.ubc.ca/)

| **Criteria for Inclusion in BC-HTC** | |
| --- | --- |
|  | |
| All individuals:   - tested at the BCCDC Public Health Laboratory (BC-PHL ) for HCV or HIV OR - reported to BC public health as a confirmed case of HCV, HIV or AIDS, HBV, or active TB OR - included in BC Enhanced Strain Surveillance System (EHSSS) as an acute HBV or HCV case | |
| All individuals meeting at least one the above criteria were linked internally across all their tests and case reports. Those with a valid personal health number (PHN) were then sent for deterministic linkage with the province-wide Cancer and Ministry of Health (MoH) datasets. | |
| **Provincial Communicable Disease Data Sources:** | **Data Date Ranges:** |
| BC-PHL HIV laboratory testing datasets (tests: ELISA, Western blot, NAAT, p24, culture) | 1988–2015 |
| BC-PHL HCV laboratory tests datasets (tests: antibody, HCV RNA, genotyping) | 1992–2019 |
| HIV/AIDS Information System (HAISYS) (public health HIV/AIDS case reports) | 1980–2015 |
| Integrated Public Health information System (iPHIS) (public health case reports of HCV, HBV, and TB) | 1990–2015 |
| Enhanced Strain Surveillance System (EHSSS) (risk factor data on a subset of acute HCV and acute HBV cases) | 2000–2013 |
| **Cancer and MoH Administrative Data Sources:** | **Data Date Ranges:** |
| BC Cancer Registry (BCCR) (primary tumour registry, excludes metastatic cancers) | 1923–2016 |
| Client Roster (CR) (Registry of enrollment in the universal public health insurance plan including residential history) ^S1^ | 1990–2016 |
| Discharge Abstracts Dataset (DAD) (hospitalization records) ^S2^ | 1985–2015 |
| Medical Services Plan (MSP) (physician diagnostic and billing data) ^S3^ | 1990–2015 |
| PharmaCare/PharmaNet (Pharma) (prescription drug dispensations) ^S4, S5^ | 1985–2019 |
| BC Vital Statistics (VS) (deaths registry)^S6^ | 1985–2019 |
| NACRS (Emergency Departments) ^S7^ | 2012–2015 |
| Chronic Disease Registry^S8^ | 1992–2015 |
| The final BC-HTC comprises all individuals successfully linked on PHN to the MoH Client Roster^S1^ (a registry of all BC residents enrolled in the publicly-funded universal healthcare system) | |

HCV: Hepatitis C Virus; HBV: Hepatitis B Virus; HIV/AIDS: Human Immunodeficiency Virus/Acquired Immunodeficiency Syndrome; BC-PHL: BC Public Health Laboratory: RNA: Ribonucleic Acid; PCR: Polymerase Chain Reaction.

| **Additional file 1: Table S2**  HCV Care cascade definitions |
| --- |
| **1. HCV Diagnosed**^†^  On or before December 31 of the year assessed:   - a confirmed anti-HCV positive test   OR   - a confirmed HCV RNA positive test   OR   - an HCV genotype result   OR   - dispensation of interferon-based or direct-acting antivirals   OR   - a confirmed public health HCV case report |
| **2. HCV RNA Tested**   - At least one HCV RNA (PCR) test on record on or before December 31 of the year assessed   OR   - an HCV genotype result on or before December 31 of the year assessed   OR   - dispensation of interferon-based or direct-acting antivirals |
| **3. HCV RNA Positive**   - At least one HCV positive RNA (PCR) test on record on or before December 31 of the year assessed.   OR   - an HCV genotype result on or before December 31 of the year assessed   OR   - dispensation of interferon-based or direct-acting antivirals - It excludes individuals with no dispensation of interferon-based or direct-acting antiviral treatment whose last HCV RNA test on record is negative (these individuals are considered to have spontaneously cleared and thus, are not actively infected/non-viraemic) |
| **4. HCV Genotyped**   - At least one valid genotype test result on record on or before December 31 of the year assessed   OR   - dispensation of interferon-based or direct-acting antivirals |
| **5. Initiated HCV Antiviral Treatment**  Dispensation of antivirals specific to HCV treatment (interferon-based or direct-acting antivirals )^‡^, on or before December 31 of the year assessed. |
| **6. SVR**^‡^   - The first HCV RNA test on record 10 weeks after the last antiviral dispensation and on or before December 31 of the year assessed is negative. If no HCV RNA test is available to estimate SVR, then SVR status is considered undetermined. |
| ^†^Earliest available laboratory testing data (1992), reportable disease data (1990), and drug dispensation data (2000)  ^‡^DINPIN: 2239730, 2241159, 2246026, 2246027, 2246028, 2418355, 2425890, 2425904, 2432226, 2246029, 2246030, 2248077, 2248078, 2253410, 2253429, 2254581, 2254603, 2254638, 2254646, 2370816, 2371448, 2371456, 2371464, 2371472, 2371553, 2416441, 2436027, 2436396, 2436418, 2436426, 2439212, 2444747, 2444755, 2447711, 2451131, 2452294, 2456370, 2467542  Abbreviations: HCV, hepatitis C virus; RNA, ribonucleic acid, PCR; polymerase chain reaction, SVR; sustained virological response, DINPIN; Drug Information Number/Product Identification Number. |

| **Additional file 1: Table S3** Covariate BC-HTC administrative code definitions |
| --- |
| **Material and Social Deprivation Quintiles (Ever)** |
| The Québec Index of Material and Social Deprivation^S9^ was calculated based on 6-digit postal code of individuals’ residence. The deprivation index combines six indicators related to health and welfare that represent material or social deprivation and are available by enumeration area in Canadian census data: 1) proportion of persons without high-school diploma 2) ratio of employment to population 3) average income 4) proportion of persons separated, divorced, widowed 5) the proportion of single-parent families 6) proportion of people living alone. |
| **Injection drug use (IDU)** |
| Injection drug use was defined at the occurrence of at least 2 physician visits, 1 hospitalization, OR 1 emergency department visit related to major drug-related diagnoses involving addiction, dependence, and drug-induced mental disorders; illicit drug use most likely to be injecting (e.g. excluding cannabis), or illicit use of prescribed drugs including: hallucinogens, barbiturates/tranquillizers, sedatives, hypnotics, anxiolytics, opioids, cocaine, amphetamine; or discharge to drug rehabilitation, counselling, and surveillance  (11 ≤ diagnosis age ≤ 65). Recent PWID was IDU between 2012 and 2015, while past was IDU before 2012.  **Physician Billing Data**: MSP ICD-9 diagnostic codes: starting with 292, 970, 3040-42, 3044-49, 3054-57, 3059, 6483, 7960, 9621, 9650, 9658, 9663-64, 9670, 9684-85, 9694, 9696-99, 9700, 9701, 9708, 9709, E8500 ,or exact codes  V6542  **Hospitalization Data**: DAD1/ICD-9-CM: starting with 292, 970, 3040-42, 3044-49, 3054-7, 3059, 6483, 7960, 9621, 9650, 9658, 9663-64, 9670, 9684-85, 9694, 9696-99, 9700, 9701, 9708, 9709, E8500 ,or exact codes  V6542; NACRS/DAD2/ICD-10-CA: starting with F11, F13-5, F19, Z722 or exact codes R781-82, T387, T400-T406, T408-09, T412, T423-28, T436, T438-39, T507; NACRS (ICD10 complaint codes): 751, 753. |
| **Problematic alcohol consumption** |
| Alcohol misuse was defined at the first occurrence of 2 MSP or 1 hospitalization codes for major alcohol-related diagnoses including alcoholic mental disorders and dependence/abuse syndromes; alcoholic polyneuropathy, myopathy, cardiomyopathy; pseudo Cushing’s syndrome; or discharge to alcohol rehabilitation, counselling, or surveillance .  **Physician Billing Data:** MSP ICD-9 diagnostic codes: starting with 291, 303, 3050, 3575, 4255  **Hospitalization Data:** DAD1/ICD-9-CM: starting with 291, 303, 3050, 3575, 4255; NACRS/DAD2/VS ICD-10-CA: starting with F10, E244, G312, G621, G721, I426, Z502, Z714 |
| **Major mental health illness** |
| Major mental illness was defined at the first occurrence of 1 hospitalization diagnostic code OR 2 MSP diagnostic codes from a psychiatrist visit for schizophrenic, bipolar, delusional, nonorganic psychotic, adjustment, anxiety, dissociative, personality and major depressive disorders.  **Physician Billing Data:** MSP ICD-9 diagnostic codes: starting with 295-298, 300-301, 308-309, 311 or exact code 50B AND claim specialty = 3  **Hospitalization Data:** DAD1/ICD-9-CM: starting with 295-298, 300-301, 308-309, 311; NACRS/DAD2/VS/ICD-10-CA: starting with F20-F25, F28-F34, F38-F45, F48, F60-F61 |
| **Hepatitis B virus (HBV) co-infection** |
| HBV coinfection was defined at the first occurrence of 2 MSP or 1 hospitalization for HBV, 2 MSP fee item codes for HBV DNA or HBV e-antigen testing, 1 dispensation for HBV-specific antiviral treatments, or a confirmed public health case report recorded in the integrated Public Health Information System (iPHIS) defined on the basis of provincial guidelines.  **Physician Billing Data:** MSP ICD-9 diagnostic codes: starting with 702, 703, V0261; Fee item codes: 90675, 90690, 90831, 91765.  **Hospitalization Data:** DAD1/ICD-9-CM: starting with 702, 703, V0261; DAD2/ICD-10-CA: starting with B16, B180, B181, Z2250.  **PharmaNet Data:** DINPIN numbers: 02239193, 02247128, 02247823, 02282224, 02288389 |
| **Human immunodeficiency virus (HIV) co-infection** |
| HIV coinfection was defined at the first occurrence of 3 MSP or 1 hospitalization for HIV, or a positive HIV serologic test, HAISYS or BC Vital Statistics indication.  **Physician Billing Data:** MSP ICD-9 diagnostic codes: starting with 042, 043, 044, 7953, 7958, 79571, V08.  **Hospitalization Data:** DAD1/ICD-9-CM: starting with 042, 043, 044, 7953, 7958, 79571, V08; DAD2/ICD-10-CA: starting with B20-B24, B9735, F024, O987, R75, Z21. |
| **Liver cirrhosis** |
| End Stage Liver Disease secondary to Cirrhosis was flagged at the first occurrence of either 1 physician visit or 1 hospitalization code relevant to decompensated cirrhosis (esophageal varices, SBP, hepatorenal syndrome, ascites, and portal hypertension) and compensated cirrhosis (chronic hepatitis failure (alcoholic/non-alcoholic), hepatic failure, unspecified, unspecified cirrhosis of the liver, other cirrhosis of the liver).  **Physician Billing Data:** MSP ICD-9 diagnostic codes: starting with 4562; exact codes 4560, 4561, 56723, 5722, 5724, 5712, 5728, 5715, 7895, 07044, 5713.  **Hospitalization Data:** NACRS/DAD1/ICD-9-CM diagnostic codes: starting with 4562; exact codes 4560, 4561, 56723, 5722, 5723, 5724, 5712, 5728, 5715, 7895, 07044, 5713.  DAD2/NACRS/VS ICD- 10-CA diagnostic codes: starting with K703; exact codes: I850, K652, K721, K729, K766, K767, K7460, K7469, R18, I98.20, I98.3, K704. |
| Abbreviations: DAD, discharge abstracts database; DIN, drug identification number; ICD, international classification of diseases; MSP, medical services plan; PIN, product information number |

**Additional file 1: Table S4** Estimated prevalence of anti-HCV positive women (including untested and undiagnosed individuals) and viremia rate for years 2012 to 2019

| Year | BC population | Diagnosed anti-HCV positive women (n) | Estimated total anti-HCV positive women (n) | Estimated prevalence of anti-HCV positive women (%) | Estimated number of viraemic women (n) | Viremia rate (%) (in BC population) | Viremia rate (%) (in anti-HCV positive women |
| --- | --- | --- | --- | --- | --- | --- | --- |
| 2012 | 4,566,769 | 18,938 | 25,251 | 0.6% | 16,250 | 0.4% | 64.4% |
| 2013 | 4,630,077 | 19,265 | 24,997 | 0.5% | 15,775 | 0.3% | 63.1% |
| 2014 | 4,707,103 | 19,613 | 24,818 | 0.5% | 15,320 | 0.3% | 61.7% |
| 2015 | 4,776,388 | 20,062 | 24,710 | 0.5% | 14,369 | 0.3% | 58.1% |
| 2016 | 4,859,250 | 19,965 | 24,069 | 0.5% | 12,982 | 0.3% | 53.9% |
| 2017 | 4,924,233 | 19,871 | 23,444 | 0.5% | 11,617 | 0.2% | 49.6% |
| 2018 | 5,001,170 | 19,703 | 22,750 | 0.5% | 9,944 | 0.2% | 43.7% |
| 2019 | 5,071,336 | 19,522 | 22,056 | 0.4% | 8,677 | 0.2% | 39.3% |

**Supplementary References**:

1. British Columbia Ministry of Health [creator]. Client Roster (Client Registry System/Enterprise Master Patient Index). British Columbia Ministry of Health [publisher]. Data Extract. MOH (2013). 2016. <https://www2.gov.bc.ca/gov/content/health/health-forms/online-services>
2. British Columbia Ministry of Health [creator]. Discharge Abstract Database (Hospital Separations). British Columbia Ministry of Health [publisher]. Data Extract. MOH (2013). 2016. <https://www2.gov.bc.ca/gov/content/health/health-forms/online-services>
3. British Columbia Ministry of Health [creator]. Medical Services Plan (MSP) Payment Information File. British Columbia Ministry of Health [publisher]. Data Extract. MOH (2013). 2016.<https://www2.gov.bc.ca/gov/content/health/health-forms/online-services>
4. British Columbia Ministry of Health [creator]. PharmaCare. British Columbia Ministry of Health [publisher]. Data Extract. MOH (2013). 2020. <https://www2.gov.bc.ca/gov/content/health/health-forms/online-services>
5. British Columbia Ministry of Health [creator]. PharmaNet. British Columbia Ministry of Health [publisher]. Data Extract. MOH (2013). 2020. <https://www2.gov.bc.ca/gov/content/health/health-forms/online-services>
6. BC Vital Statistics Agency [creator]. Vital Statistics Deaths. BC Vital Statistics Agency [publisher]. Data Extract. BC Vital Statistics Agency (2014). 2020. <https://www2.gov.bc.ca/gov/content/health/health-forms/online-services>
7. British Columbia Ministry of Health [creator]. National Ambulatory Care Reporting System. British Columbia Ministry of Health [publisher]. Data Extract. MOH (2017). 2017

<https://www2.gov.bc.ca/gov/content/health/health-forms/online-services>

1. British Columbia Ministry of Health [creator]. Chronic Disease Registry. British Columbia Ministry of Health [publisher]. Data Extract. MOH. (2017) 2017.

<https://www2.gov.bc.ca/gov/content/health/health-forms/online-services>

S9. Pampalon R, Hamel D, Gamache P, Raymond G. A deprivation index for health planning in Canada. Chronic Dis Can 2009; 29(4): 178-91.
